# Singleton Pregnancy Outcomes after In Vitro Fertilization with Fresh or Frozen-Thawed Embryo Transfer and Incidence of Placenta Praevia

**DOI:** 10.1155/2014/431797

**Published:** 2014-04-13

**Authors:** Sara Korosec, Helena Ban Frangez, Ivan Verdenik, Urska Kladnik, Vanja Kotar, Irma Virant-Klun, Eda Vrtacnik Bokal

**Affiliations:** Department of Human Reproduction, Division of Gynaecology, University Medical Centre Ljubljana, Šlajmerjeva 3, SI-1000 Ljubljana, Slovenia

## Abstract

The aim of the study was to compare the single pregnancy and neonate outcome after fresh and frozen-thawed embryo transfer in the in vitro fertilization programme (IVF). The study focused on clinical and laboratory factors affecting the abnormal placentation, especially placenta praevia, in patients conceiving in the IVF programme. The results confirm that neonates born after frozen-thawed embryo transfer had significantly higher mean birth weight than after fresh embryo transfer (ET). Moreover, the birth weight distribution in singletons was found to shift towards “large for gestation” (LGA) after frozen-thawed ET. On the other hand, the pregnancies after fresh ET were characterized by a higher incidence of placenta praevia and 3rd trimester bleeding. Placenta praevia was more common in IVF patients with fresh ET in a stimulated cycle than in patients with ET in a spontaneous cycle. It occurred more frequently in patients with transfer of 2 embryos. From this point of view, single ET and ET in a spontaneous cycle should be encouraged in good prognosis patients in the future with more than two good quality embryos developed. An important issue arose of how the ovarian hormonal stimulation relates to abnormal placentation and if the serum hormone levels interfere with in the IVF treatment results.

## 1. Introduction

The reduction in number of embryos transferred into the uterus is crucial in preventing multiple pregnancies, one of the major in vitro fertilization (IVF) complications, since it is known that they carry increased risk for maternal and neonatal morbidity in comparison to singleton pregnancies [1–3]. Frozen-thawed embryo transfer (ET) technique enabled a transfer of fewer embryos into the uterus and storage of surplus embryos by cryopreservation for future use [3, 4]. The consequence of such “preventing multiplicity” practice is a rapidly increasing number of frozen embryos, followed by an increased percentage of births and neonates following such infertility treatment [2–5]. It is vital to confirm that newborns born after frozen-thawed ET are at least as healthy as the newborns born from fresh ET procedures [3, 5–12].

Until now no significant differences regarding children's outcome have been established in comparison to fresh ET since the worldwide introduction of frozen-thawed ET into the everyday clinical practice [5–8, 10, 11, 13]. The studies have shown that neonatal outcome after frozen-thawed ET is comparable to the outcome after fresh ET [3, 5–11, 13]. Even though there were no major differences found in the outcome, it was nevertheless indicated that neonates born after frozen-thawed ET seem to have a higher birth weight. Furthermore, there was a decline in placenta praevia and antepartum haemorrhage after frozen-thawed ET in comparison to fresh ET [5, 8–13].

Placenta praevia and certain other placental abnormalities, followed by antepartum haemorrhage, generally occur more often in the IVF pregnancies and the causes are still poorly understood [2, 9, 12, 14, 15]. It has been speculated that placenta praevia more often develops after an IVF treatment because embryos are transferred via vagina and cervix [14]; however, a more recent study on gamete intrafallopian transfer (GIFT) showed a similar rate of placenta praevia and we can therefore establish that the association between placenta praevia and ET procedure is less probable [12]. Other placentation abnormalities with an increased incidence in IVF pregnancies are placenta accreta [16, 17], vasa praevia [18, 19], and abnormal umbilical cord insertion [20]. Some of the crucial IVF pregnancy complications, such as intrauterine growth restriction (IUGR), low birth weight, and premature delivery [2, 3, 10, 21–23], could, to some extent, be the consequences of the above-mentioned placental abnormalities [2, 14, 17].

The aim of the study was to evaluate the differences between the singleton pregnancy outcomes after fresh or frozen-thawed ET in the IVF programme. The additional aim was to identify possible connections of placenta previa to clinical parameters related to IVF procedure (including stimulation protocol, number of oocytes, number of developed and transferred embryos, and embryo quality), since the phenomenon of increased placenta praevia rates in IVF-derived pregnancies is still poorly understood.

## 2. Material and Methods

We analyzed the differences between pregnancy outcomes after fresh and frozen-thawed ET in our population of patients. Additionally, we analyzed the connections of placenta praevia to clinical parameters in women who conceived after in vitro fertilization procedure at our department.

### 2.1. Patients

In this retrospective cohort study we analyzed the outcomes of 1126 singleton pregnancies after IVF-ET, 211 of which have resulted from frozen-thawed ET and 915 pregnancies from fresh ET. The IVF-ET procedures were performed at the Department of Human Reproduction, University Medical Centre Ljubljana, in the time period between January 2004 and December 2011.

### 2.2. Ovarian Stimulation

Short antagonist cetrorelix protocol (Cetrotide: Serono, London, UK, or Asta Medica AG, Frankfurt, Germany) or long desensitization buserelin protocol (Suprefact; Hoechst AG, Frankfurt/Main, Germany) was used for ovarian stimulation. The short protocol consisted of GnRH antagonist cetrorelix acetate in a dose of 0.25 mg being administered at the time; the dominant follicle measured ≥14 mm in diameter after daily stimulation with 225 IU of follicle stimulating hormone (Gonal-F: Serono, Switzerland, Puregon) from day 2 of the menstrual cycle. In the long protocol buserelin was used until the criteria for ovarian desensitization were fulfilled (estradiol <0.05 nmol/L, follicles <5 mm in diameter) from day 22 of the menstrual cycle at a daily dose of 0.6 mL (600 pg) subcutaneously. Afterwards, 225 IU of follicle stimulating hormone was administered daily. All patients were administered 10,000 IU of human chorionic gonadotrophin (hCG) (Pregnyl; N.V. Organon) when at least three follicles were measured ≥17 mm in diameter.

### 2.3. Oocyte Retrieval, Fertilization, and Embryo Culture and Transfer

Transvaginal ultrasound-guided oocyte retrieval was performed 34 hours after hCG administration and under local anesthesia. Fertilization of oocytes was performed using classical in vitro fertilization (IVF) or intracytoplasmic sperm injection (ICSI).

Embryos were cultured to the blastocyst stage in sequential media M1 and M2 (Origio/MediCult, Jyllinge, Denmark) until day 5. One or two best quality blastocysts were transferred in the uterus on day 5. The blastocyst grading system introduced by Gardner et al. [24] was used to evaluate the blastocyst quality. Supernumerary blastocysts were frozen on day 5 or day 6 using our original modified two-step Menezo method described by Virant-Klun et al. [25] and stored in liquid nitrogen at −196°C. We rarely performed day 2 or day 3 ET of cleavage stage embryos in those patients with previous blastocyst development failure and only one or two embryos developed.

When needed, the cryopreserved blastocysts were thawed on the day of the transfer. Coordination between the endometrium and blastocysts in the frozen-thawed cycles was performed as described in Virant-Klun et al. [25]. One or two survived blastocysts with more than 50% of nondamaged cells were transferred 4 days after the disappearance of the dominant follicle in the natural cycle in women with regular menstrual patterns. In women with irregular menstrual cycles a minimal stimulation with 75 IU of rFSH was started daily from day 7 onward and the ET of one or two survived blastocysts was performed 6 days after the induction of ovulation with 5000 IU of hCG.

Luteal phase support consisted of 5000 IU of hCG on the day of ET in women receiving ovarian stimulation. Alternatively, 3 × 200 mg daily doses of progesterone were given intravaginally (Utrogestan: Viatris Pharma, Paris, France) until a gestational sac with fetal heart activity was visible on day 30 after the ET procedure. Pregnancies (PR) were diagnosed by beta hCG serum determinations 14 days after hCG administration and by a vaginal ultrasound examination 4 weeks after the ET. Clinical PR was defined by an ultrasound observation of a positive heartbeat on day 30 after the ET procedure.

### 2.4. Data Collection

Data on the IVF procedures were collected in the IVF laboratory at our department on a daily basis. All 14 Slovenian maternity hospitals systematically collect data on maternal demographic characteristics, medical, gynecological and reproductive history, prenatal care, pregnancy, delivery, postpartum period, and neonates for each mother-infant pair using the same definitions of variables and the same form of medical record. The data are sent to the National Institute of Public Health of the Republic of Slovenia by default and were available for this research.

The data obtained from NPIS (National Perinatal Information System) were depersonalized in order to ensure the anonymity of the women and neonates. The research was performed according to the Personal Data Protection Act (UL). The research was approved by the National Medical Ethics Committee.

Slovene reference standard curves for weight, length, and head circumference at birth for given gestational age were used for fetal and neonatal growth estimation [26]. LGA (“large for gestational age”) was defined as growth above the 95th percentile and SGA (“small for gestational age”) was defined as growth below the 5th percentile according to the aforementioned population growth curves.

Preeclampsia was defined as arterial hypertension of at least 140/90 mm Hg detected for the first time in a pregnancy of at least 20 weeks or higher (measured at least 2 times and at least 6 hours apart). Additionally, at least 300 mg of proteins in daily urine collection must be present.

First trimester bleeding was defined as bleeding in the first 12 weeks of pregnancy, measured from the first day of the last menstrual bleeding (or calculated as 10 weeks after oocyte collection in women without menstruation), second trimester bleeding was defined as bleeding from 13 to 24 weeks of pregnancy, and third trimester bleeding was the one occurring after fulfilled 25 weeks of pregnancy.

Placenta praevia was diagnosed in cases of placental edge lying closer than 2 cm to the internal cervical os or over it. Placenta accreta was reported to the NPIS by the obstetrician who was performing a manual placenta removal, a postpartum abrasion, a caesarian section, or sometimes a removal of placental polyp. The report is written directly after the surgical procedure by the surgeon involved in the procedure.

### 2.5. Statistics

Statistical analysis was performed using IBM SPSS Statistics, version 19 (IBM Corporation, Armonk, NY). Student's *t*-test was used to compare normally distributed parametrical variables, Mann-Whitney's test was used to compare non-normally distributed variables, and Chi-square test was used to compare categorical variables. A multivariate logistic regression was used to identify independent risk factors. *P* value of < 0.05 was considered significant.

## 3. Results 

The patients' mean age was 33.4 ± 3.9 years. Among these patients, 653 (58%) women were treated because of female factor infertility, 202 (18%) women because of male factor infertility, and 271 (24%) women because of both female and male factor infertility.

### 3.1. Comparison of Pregnancy Outcomes after Fresh and Frozen-Thawed ET

#### 3.1.1. General

The patients' mean age, parity, previous abortions, number of embryos transferred, number of blastocyst transfers, and number of previous IVF attempts are presented in Table 1.

The mothers in the frozen-thawed ET group were significantly more often multiparous with significantly more abortions in the history than the mothers in the fresh ET group. They also had more blastocyst transfers and less previous IVF attempts.

#### 3.1.2. Birth Characteristics and Neonate Outcome

The mean birth weight of neonates, born after frozen-thawed ET, was significantly higher than the birth weight in the fresh ET group, whereas other birth characteristics and neonate outcomes were similar in both groups. The results are shown in Table 2.

Birth weight of neonates born after frozen-thawed ET was significantly higher than the birth weight of neonates born after fresh ET. Since the difference in parity between the groups could have an influence on the difference in birth weight, we performed a multivariate analysis of parity and gestational weight. The birth weight showed to be an independent variable after the implementation of logistic regression and remained significantly higher in singletons after frozen-thawed ET (*P* = 0.002).

Analysis of birth weight distributions in both groups of neonates revealed a shift of singletons after frozen-thawed ET towards the LGA. There were 10.5% of neonates after the frozen-thawed ET and 5.0% of neonates after the fresh ET who were above the 95th centile of weight for a certain gestation the difference being significant (*P* = 0.003). In addition, there were significantly less SGA pregnancies in the frozen-thawed ET in comparison to the fresh ET group (0.9% versus 3.7%, resp., *P* = 0.048).

#### 3.1.3. Abnormal Placenta and Complications during Pregnancy

Pregnancies after fresh ET were characterized by significantly higher rates of placenta praevia and 3rd trimester bleeding, as can be seen in Table 3. After frozen-thawed ET we did not find any cases of placenta praevia and 3rd trimester bleeding.

Placenta praevia was the only pathology that differed significantly between the two groups.

We analyzed the occurrence of placenta praevia in primiparous and multiparous patients in both ET groups to establish the influence of parity on the rate of placenta praevia cases. There were significantly less pregnancies with placenta praevia in the group of pregnancies resulting from frozen-thawed ET in multiparous women (4.3% versus 0%; *P* = 0.049) as well as in primiparous women (3.2% versus 0%; *P* = 0.020).

### 3.2. Parameters Related to Abnormal Placentation regardless of Embryo Transfer Type

We performed additional analysis of all pregnancies with placenta praevia in order to establish possible background factors that could be connected to pathogenesis. There were no significant differences in the number of oocytes retrieved, oocytes fertilized, and day of ET or embryo quality between IVF cycles of patients with and without placenta praevia (see Table 4).

Mean number of oocytes retrieved in fresh cycles with and without placenta praevia was similar (9.7 ± 5.5 oocytes versus 9.2 ± 5.2 oocytes, resp., *P* = 0.830).

To perform a multivariate analysis of abnormal placentation we included all cases of placenta praevia, accreta, and retained placenta that together formed a larger group of 99 pregnancies. Using logistic regression we examined connection between background parameters (maternal age, parity, uterine surgery, supernumerary embryos, number of embryos transferred, and body mass index (BMI)) and abnormal placentation.

There was no connection of abnormal placentation to maternal age, parity, uterine surgery, supernumerary embryos, or BMI. Transfer of 2 embryos was a significant factor for occurrence of abnormal placentation and spontaneous cycle ET (i.e., ET without any stimulation) was of borderline significance (*P* value being between 0.050 and 0.100) for absence of abnormal placentation. Results are shown in Table 5.

The process of comparing the groups of women with and without abnormal placentation showed no difference in terms of previous caesarean sections (CS), the type of the fertilization procedure (IVF, ICSI/TESE), or number of retrieved oocytes in the IVF cycle. The results are shown in Table 6.

## 4. Discussion

The results of this study showed that the singletons after frozen-thawed ET had significantly higher mean birth weight than the singletons after fresh ET. Moreover, the birth weight distribution in singletons after frozen-thawed ET was found to shift towards LGA. On the other hand, the pregnancies following fresh ET were characterized by a higher incidence of placenta praevia and 3rd trimester bleedings than the pregnancies after frozen-thawed ET. The abnormal placentation rates were unrelated to most IVF-associated parameters, apart from the double blastocyst transfer and transfer in a spontaneous cycle.

We performed multivariate analysis in order to establish whether the disproportion in parity influenced the difference in birth weights between the frozen-thawed and fresh ET neonates. The birth weight of frozen-thawed ET neonates remained significantly higher than the birth weight of fresh ET neonates, which is consistent with the results found in the literature [5, 7–10, 13, 22, 27]. Birth weight of frozen-thawed ET neonates proved to be a parity-independent variable with a significant shift towards LGA, which has also been discovered by other studies [9, 13, 22, 27]. The delivery of a LGA neonate represents a greater morbidity risk for the mother and neonate [28], yet another concern is the possible hidden epigenetic pathology. It is known that some rare epigenetic disorders occur more often after ART than after spontaneous conception, such as Beckwith-Wiedemann syndrome, Angelman syndrome, and possibly cancer [29, 30]. High incidence of “large offspring syndrome” has been noticed following ART of cattle and sheep resulting in high birth weight which has recently been proven to be epigenetically and phenotypically similar to Beckwith-Wiedemann's syndrome in humans [29, 30]. However, no such pathology has been observed in our population, nor in the whole Danish population [31]; therefore, other causes should also be considered [9, 13, 29]. The SGA and LBW rates were significantly reduced in the frozen-thawed ET group, thus providing us with similar results as observed by other authors [8–10, 13, 22].

The study revealed significant differences regarding abnormal placentation that more often occurs in pregnancies following ART and mostly for unknown reasons [2, 3, 9, 12, 14, 19, 32–34]. It was established that IVF conception substantially increases the risk for early preeclampsia cases [32], which is often explained by primiparity of the IVF patients. The rates of preeclampsia in our study were three times lower in the frozen-thawed ET group (*P* = 0.098) than in the fresh ET group, which is the opposite of the results obtained in a national Swedish study [9]. Our findings can be explained by a higher rate of multiparous mothers in frozen-thawed ET group, while the Swedish results remained unclarified [9].

Similarly, placenta praevia, accreta, and vasa praevia more often occur in pregnancies derived from an IVF procedure [2, 12, 14, 17, 19] and with unfavorable consequences [15]. The placenta praevia rates in our and other reports are higher in multiparous women [15] but lower in the frozen-thawed ET group [9, 12]. Since in frozen-thawed ET group there were significantly more multiparous women, but significantly lower placenta praevia rates, parity could not in any case lower the placenta praevia rate in this group of pregnancies. On the other hand, there was no difference in placenta accreta, manually removed placenta, and retained placenta cases between the frozen-thawed and fresh ET groups in our study.

Australian report discovered less haemorrhages in pregnancies after frozen-thawed and spontaneous cycle ET [12]. Israeli investigators found the connection between abnormal placentation and higher estradiol serum levels [17]. Interestingly, there were no variations between oocytes retrieved in cycles with and without placenta praevia in our study that would indicate high ovarian response being the risk factor for placenta praevia in general. Our study was larger than the Israeli study (32 cases of placenta praevia versus 2 cases, resp.); however, we did not include the serum estradiol measurements into the analysis. Since we did not find any cases of placenta praevia in pregnancies after ET in a spontaneous cycle, we can conclude that the hormonal stimulation might have influenced the manifestation of placenta praevia. With reference to our and other studies [10, 12, 17, 22], the effect of hormonal stimulation, including the estradiol levels at the time of implantation, needs to be investigated further.

The manifestation of placenta praevia was significantly related to the transfer of two embryos, all of them followed day 5 ET. However, there were 95% transfers of blastocysts in this period in our IVF programme, since we adopted the blastocyst transfer policy early. Therefore the impact of blastocyst transfer versus cleavage stage embryos on the pregnancy outcome is difficult to evaluate in this patient population. The Swedish analysis has associated placenta praevia with the blastocyst transfer [35]. Contrastingly, a recent large Japanese study (over 270.000 single ET) established that the blastocyst transfer was not associated with placental abnormalities, including placenta praevia; however, frozen-thawed ET was indeed associated with the risk of placenta accreta. [27]. In a recent meta-analysis, which did not include the study mentioned above, blastocyst transfer was linked to higher risk of preterm birth [11], while the Japanese study revealed reduced low birth weight rates and increased LGA weight after transferring blastocysts [27].

In contrast to expected risk factors for placenta praevia observed in the general population, previous caesarean section and uterine surgery were not connected to abnormal placentation in our study population. Similarly, higher parity and abortion rates were not risk factors for placenta praevia in pregnancies following frozen-thawed ET.

Our current study points to the fact that abnormal placentation more commonly occurs in IVF patients with fresh ET in a stimulated cycle and it is more common among patients with the transfer of 2 embryos. From this point of view, single ET and ET in a spontaneous cycle should be encouraged. It seems that the reasons for placenta praevia in the IVF programme differ from those found in the general population, which are caesarean sections, multiparity, or uterine surgery; all these factors did not significantly influence the placentation in our IVF population of patients.

## 5. Conclusion

The results of our study revealed significantly more LGA neonates in pregnancies after frozen-thawed ET in comparison to fresh ET outcome. Does the higher birth weight indicate a more healthy pregnancy, or is this birth weight shift pathological, carrying all the risks of LGA, metabolic disorders, or even epigenetic changes? Placenta praevia seems to be a complication related to stimulated cycles with 2 fresh embryos transferred. The known risk factors for placenta praevia in the general population were not significant in our study population and we may therefore conclude that the patients conceiving in the IVF programme represent a special population. Such investigations give us the opportunity to research the possible background of still poorly understood abnormal placentation. Better results after frozen-thawed ET and ET in a spontaneous cycle imply that the environment at the moment of ET is important and may influence the pregnancy outcome. How does the hormonal stimulation relate to these pathologies? It is not excluded that IVF pregnancies possess somehow altered mechanisms of placentation, foetal development, and growth.

## Figures and Tables

**Table 1:** Clinical characteristics of patients having fresh and frozen-thawed ET.

	Frozen-thawed ET Number of cases (%)	Fresh ET Number of cases (%)	*P* value
Number of patients	**211**	**915**	
Mean age (years ± SD)	33.5 ± 3.7	33.4 ± 4.0	0.724
Multiparous	80 (39.7)	232 (25.4)	*<0.001
Previous abortions (mean, range)	0.46 (0–4)	0.33 (0–6)	*0.011
Single ET	51 (24.2)	164 (17.9)	*0.041
Double ET	160 (75.8)	751 (82.1)	
Blastocyst transfer	194 (91.9)	802 (87.5)	
Double blastocyst transfer	142 (67.3)	454 (49.6)	
Previous IVF attempts (mean ± SD)	1.89 ± 1.2	2.19 ± 2.1	*0.03
1 attempt	117 (55.5)	406 (44.5)	
2 attempts	46 (21.8)	248 (27.2)	
3 attempts	32 (15.2)	112 (12.3)	
4 attempts	9 (4.3)	73 (8.0)	
5 attempts	2 (0.9)	29 (3.2)	
6 attempts	3 (1.4)	15 (1.6)	
7 attempts	2 (0.9)	11 (1.2)	
8 and more attempts	0	19 (2.2)	
Infertility cause			
Tubal	52 (24.6)	253 (27.7)	0.511
Endometriosis	43 (20.3)	187 (20.4)	0.982
Endocrine	44 (20.9)	198 (21.6)	0.931
Male	39 (18.5)	166 (18.1)	
Others**	33 (15.6)	111 (12.1)	0.277

*Statistically significant (*P* < 0.05), as revealed by Chi-square or Mann-Whitney test. **Other causes include oncologic patients and idiopathic infertility.

**Table 2:** Comparison of birth characteristics and neonate outcome following frozen-thawed and fresh ET.

	Frozen-thawed ET *N* = 211	Fresh ET *N* = 915	*P* value
Birth weight (g ± SD)	3354.9 ± 626.3	3200.1 ± 632.7	*0.001
Mean gestation age (weeks ± SD)	38.6 ± 2.59	38.5 ± 2.51	0.558
Preterm birth** (%)	9.0	12.1	0.232
Foetal distress (%)	3.3	3.7	1.000
Caesarean section (%)	25.1	25.8	0.930
Admittance to neonatal intensive care unit (%)	5.7	7.4	0.458
Low (0–6) 5-minute Apgar score (%)	1.4	1.6	1.000
Congenital malformation (%)	6.2	7.4	0.657
Chromosomal malformation (%)	0	0.1	1.000

*Statistically significant (*P* < 0.05), as revealed by *t*-test. **Preterm birth—birth before 37 weeks of gestation.

**Table 3:** Comparison of placental and amniotic abnormalities between pregnancies after frozen-thawed and fresh ET.

	Frozen-thawed ET *N* = 211 Number of cases (%)	Fresh ET *N* = 915 Number of cases (%)	*P* value
Preeclampsia	2 (0.9)	28 (3.1)	0.098
1st trimester bleeding	14 (6.6)	74 (8.1)	0.570
2nd trimester bleeding	2 (0.9)	27 (2.9)	0.144
3rd trimester bleeding	0 (0.0)	18 (2.0)	0.034*
Polihydramnion	2 (0.9)	7 (0.8)	0.679
Oligohydramnion	3 (1.4)	18 (2.0)	0.781
Placenta accreta	7 (3.3)	29 (3.2)	0.831
Placenta praevia	0 (0.0)	32 (3.5)	0.002*
Retained placenta	10 (4.7)	34 (3.7)	0.553
Manual removal of placenta	11 (5.2)	51 (5.6)	1.000

*Statistically significant (*P* < 0.05), as revealed by Chi-square.

**Table 4:** IVF-ET cycle characteristics resulting in pregnancies with and without placenta praevia.

	Pregnancies with placenta praevia *N* = 32	Pregnancies without placenta praevia *N* = 1095	*P*
Day of ET			
2	0	14 (1.3%)	0.775
3	0	9 (0.8%)	
4	0	1 (<0.1%)	
5	32 (100%)	1037 (94.7%)	
6	0	34 (3.1%)	
Number of morulae and blastocysts on day 5			
2 or 3	18 (56%)	610 (56%)	0.410
4 or 5	10 (31%)	258 (23%)	
6 or more	3 (3%)	222 (21%)	
ET number (1 or 2) and embryo quality (poor/good)**		∗	
1—poor embryo	0	13 (1%)	0.491
2—poor/poor embryo	3 (9%)	76 (7%)	
1—good embryo	6 (19%)	153 (14%)	
2—good/poor embryo	2 (6%)	197 (18%)	
2—good/good embryo	21 (66%)	461 (42%)	
Stimulation protocol		∗	
Long protocol	18 (56%)	685 (63%)	0.403
Short protocol	14 (44%)	359 (33%)	

*24 data on stimulation in a group of pregnancies without placenta praevia missing and 18 data on embryo quality in group without placenta praevia missing.

**Good quality: good blastocysts quality according to Gardner criteria [24] on day 5 or 6, cleavage stage embryo with little (<10%) or no fragmentation on day 2 or 3, and equal (round) blastomeres on day 2 or 3. Poor quality: embryos not reaching the criteria for good quality.

**Table 5:** Multivariate analysis using logistic regression showing connection of abnormal placentation to background parameters in 99 women.

Parameter	*P*	Odds ratio (OR)	95% confidence interval (CI)
Maternal age	0.846	0.995	0.943–1.050
Multiparity	0.293	0.751	0.459–1.235
Previous uterine surgery (including myomectomy, caesarean section, reconstructive surgery, or septum resection)	0.490	1.132	0.718–1.784
BMI			
Extremely low (under 18.5)	0.402	0.538	0.126–2.290
High (above 30)	0.544	0.764	0.320–1.823
Supernumerary embryos developed in IVF cycle	0.275	1.269	0.827–1.946
Transfer of 2 embryos	0.043	1.952	0.020–3.735
Spontaneous cycle ET	0.064	0.417	0.150–1.164

**Table 6:** Previous caesarean sections, number of oocytes retrieved, and fertilization procedure in women with and without abnormal placenta.

Parameter	Pregnancies with abnormal placenta *N* = 99 *N* (%)	Pregnancies without abnormal placenta *N* = 1028 *N* (%)	*P*
Previous CS	4 (4)	31 (3.1)	0.538
Number of oocytes retrieved (mean ± SD)	10.0 ± 5.7	10.0 ± 5.6	0.687
IVF	54 (54.6)	591 (57.5)	
ICSI/TESE	44 (44.4)	417 (40.6)	
	1 (1.0)	20 (1.9)	0.671
